# Memoirs of the Royal Medical Society at Paris, Vol. II

**Published:** 1783

**Authors:** 


III. *Memoirs of the Royal Medical Society at Paris, Vol. II.* *(Continued from page* 153*).*

SURGERY.—1. *Experiments with fixed air in ſurgical diſeaſes. By* M. de Lalouette, jun.—We have here an account of the effects of this application in fourteen caſes of ulcers. Our author has obſerved that it often increaſes the growth of fungus. In ulcers accompanied with inflaminflammation, he has seen it do harm by increasing the inflammation and lessening the discharge. The only cases in which he found it useful were pale, livid ulcers with a copious suppuration.—2. *An account of the Cæsarian operation successfully performed on a lady* 28 *years old, August* 31, 1778, *by* M. Hennequin, surgeon at Charleville.—The patient of whom we have here an account was short, and in her first pregnancy. She was taken in labour on the 29th of August. The elbow of the child presented itself, and our author attempted in vain to introduce his hand into the uterus. On the 31st he performed the section, on the right side of the abdomen several fingers breadth from the linea alba. The external incision was eight inches long, that of the peritonæum and uterus about seven. Only a very few ounces of blood were lost in the operation, and a dead fœtus, of a larger size than usual, with its placenta, were extracted, and the uterus cleared of its coagulated blood. The omentum was next reduced, and the lips of the wound brought together by means of three sutures. A journal of the case is given to the 24th of September. From the 20th to the 24th day after the operation pus flowed from the vagina. On the 18th of October the menses apappeared and continued 24 hours. Nov. 1ſt. the wound was ſo completely cicatrized that the patient went to church, and has continued well ever ſince.—3. *Caſes of extravaſation of blood into the bladder.* *By* M. de la Perche, jun. M.D. at Tonneins in Guienne.—A man in his 77th year, who for 15 years had been ſubject to retention of urine, and obliged to make uſe of a catheter, one day complained of great pain, ſucceeded by fever, diſtention of the bladder, and ſwelling of the hæmorrhoidal veins. The catheter afforded him no relief, as no urine flowed through it, but on withdrawing it, the holes at its extremity were found filled with coagulated blood. Our author, who was conſulted on the occaſion, aſcribed theſe ſymptoms to an hæmorrhage of the bladder. Blood which comes from the kidneys, he obſerves, mixes with the urine without coagulating; while that which iſſues from the neck of the bladder conſtantly coagulates. After trying a variety of remedies without effect, he recollected a paſſage in Houllier, where we are told, that *ubi grumeſcit ſanguis ſummum eſt periculum, etſi medicamentis non ceſſerit, inciſo perinæo molienda curatio eſt, ad vitandam corruptionem et mortem.* But in this caſe the parts ſeemed to be too much inflamed to allow of

ſuch

ſuch an operation. He therefore conceived the idea of drawing out the coagulated blood by ſuction. For this purpoſe a catheter was introduced, to which he adapted a ſmall ſyringe ſurrounded with wax to prevent communication with the outer air. He then began to pump, and the firſt ſtroke filled the catheter with grumous blood. This operation was repeated till at length bloody urine began to flow through the catheter, and then the pain abated, and the patient recovered.—With this apparatus the catheter was obliged to be removed every time; but he has ſince improved it by cutting off an inch and a half of the handle of a catheter, and fixing this piece as a pipe to a ſyringe. In the place of the piece cut off, he has ſodered to the catheter a tube of the ſame length and metal as the portion cut off, but large enough to admit the whole of the new canula, by which means a cylindrical tube is formed from the bladder to the body of the ſyringe which becomes filled with blood, and may be emptied without withdrawing the catheter. A ſecond caſe of the ſame kind is related by our author ſucceſsfully treated in the ſame manner. A third caſe of a ſimilar nature has been communicated to the ſociety by M. Hallé, in which M. de la Perche's

 method method ſucceeded after every other had failed, and after warm water injected into the bladder had ſo diſtended it as to occaſion exquiſite pain to the patient.—4. *Miſcellaneous Obſervations.* Under this head are given conciſe abſtracts of papers communicated to the ſociety on the following ſubjects, viz. 1. *On the internal uſe of aſſafœtida in Caries, by* M. Beerenbrock. This is a ſhort account of a mode of treatment recommended by Mr. Theden, a Pruſſian ſurgeon. From two to ſix ſcruples of aſſafœtida are directed to be given every day.—2. *On the operation of Lithotomy at two different times, by* M. Beauvais de Preau, ſurgeon at Orleans.—Two caſes are related; the firſt is that of a girl ſixteen years old, who for two years had experienced ſymptoms of the ſtone. The author made his inciſion through the urethra, and with his forceps got hold of the ſtone, but it reſiſted ſo much, that on a ſuppoſition of its adhering to the bladder he reſolved to wait the event of a ſuppuration. At the end of three days the ſtone preſented itſelf at the wound, and was eaſily extracted. The patient recovered.—The ſubject of the other caſe was a man aged fifty-ſeven. In this patient a ſimilar difficulty occurred in the extraction of the ſtone, and it was dedeferred in the ſame manner for two days, when it was taken out with the fingers.—3. *A caſe of imperforated uterus, by* M. Rathieu, ſurgeon at Langres.—A girl 18 years old complained of a pain of the back and loins, and about the region of the uterus, and likewiſe of a head-ache, laſſitude, want of ſleep, and other ſymptoms of obſtructed menſes. Her countenance was florid, blood flowed occaſionally from her noſtrils, and the hypogaſtric region appeared to be ſwelled. On examination the uterus was found to fill a great part of the abdominal cavity. The ſurgeon ſuppoſing this diſtenſion to be owing to the imperforation of the uterus, ventured to puncture it. By this operation three quarts of blood were diſcharged, and the patient at firſt ſeemed to be relieved, but a fatal inflammation ſoon followed. On diſſection the ovaries appeared to be in a natural ſtate, and the uterus was found contracted as it uſually is about the ſixth day after delivery, and filled with a coagulum of blood.—4. *Caſe of a ſeparation of the oſſa pubis, by* M. Hennequier of Charleville.—We have here an account of a woman 28 years old, who in a difficult labour experienced a ſeparation of the oſſa pubis of near an inch and an half in extent, and likewiſe of the ilium and ſacrum. The ſeparation was ſudden, and attended with a craſh. The patient recovered, but was a long time before ſhe was able to walk.——5. *Remarks on infuſive ſurgery, by* M. Regnaudot, phyſician at Gaudaloupe.—Etmuller in his *Diſſert. de Chirug. Infuſor.* has fully treated this ſubject. There was a time when almoſt every diſeaſe was thought capable of being cured in this way. M. Regnaudot fancied it might be uſeful in caſes where the ſtomach is affected, or in aſphyxies. He has tried it repeatedly but without ſucceſs. The mildeſt fluid, ſuch as a ſolution of gum arabic, excited fever, ſweating, ſtools, and vomiting. Infuſion of ſena conſtantly produced vomiting. The operation was performed with a ſmall ſyringe ſimilar to that uſed for injecting the lachrymal ducts. A vein was firſt laid bare and punctured, and then this ſyringe was introduced at the opening.

[*To be continued.*]

