# Errata

**Published:** 1783

**Authors:** 


					p. 243, I. 23, for M. Hennequier
read M. Hennequm ;

				

